# Harbor networks as introduction gateways: contrasting distribution patterns of native and introduced ascidians

**DOI:** 10.1007/s10530-014-0821-z

**Published:** 2014-12-10

**Authors:** Susanna López-Legentil, Miquel L. Legentil, Patrick M. Erwin, Xavier Turon

**Affiliations:** 1 Department of Biology and Marine Biology, Center for Marine Science, University of North Carolina Wilmington, 5600 Marvin K. Moss Lane, Wilmington, NC 28409 USA; 2 Departament de Biologia Animal, Universitat de Barcelona (UB), Diagonal Avenue 643, 08028 Barcelona, Spain; 3 Center for Advanced Studies of Blanes (CEAB-CSIC), Accés Cala S. Francesc 14, 17300 Blanes, Girona, Spain

**Keywords:** Tunicates, Introduced species, Barcoding, Artificial substrates, Distribution, Diversity

## Abstract

Harbors and marinas are well known gateways for species introductions in marine environments but little work has been done to ascertain relationships between species diversity, harbor type, and geographic distance to uncover patterns of secondary spread. Here, we sampled ascidians from 32 harbors along ca. 300 km of the NW Mediterranean coast and investigated patterns of distribution and spread related to harbor type (marina, fishing, commercial) and geographic location using multivariate techniques. In total, 28 ascidians were identified at the species level and another 9 at the genus level based on morphology and genetic barcoding. Eight species were assigned to introduced forms, 15 were given native status and 5 were classified as cryptogenic. *Aplidium accarense* was reported for the first time in the Mediterranean Sea and was especially abundant in 23 of the harbors. Introduced and cryptogenic species were abundant in most of the surveyed harbors, while native forms were rare and restricted to a few harbors. Significant differences in the distribution of ascidians according to harbor type and latitudinal position were observed. These differences were due to the distribution of introduced species. We obtained a significant correlation between geographic distance and ascidian composition, indicating that closely located harbors shared more ascidian species among them. This study showed that harbors act as dispersal strongholds for introduced species, with native species only appearing sporadically, and that harbor type and geographic location should also be considered when developing management plans to constrain the spread of non-indigenous species in highly urbanized coastlines.

**Electronic supplementary material:**

The online version of this article (doi:10.1007/s10530-014-0821-z) contains supplementary material, which is available to authorized users.

## Introduction

Maritime activity has been spreading non-native species around the globe since early attempts to voyage by sea (Hewitt et al. 2009). However, recent increases in the number of artificial substrates available to non-indigenous species have greatly accelerated the introduction process (Glasby and Connell 1999). Once a species is well established in a new area, local fishing and recreational boating potentially facilitate further range expansion (Wasson et al. 2001; Darbyson et al. 2009; Davidscon et al. 2010). Thus, harbors and marinas play crucial roles in the introduction of marine species, including the initial inoculation of a species from another area and the subsequent spread at a local level (also called pre-border and post-border processes; Forrest et al. 2009). To date, most studies have focused on cataloguing the exotic species observed in a given location or harbor (e.g. Arenas et al. 2006; Callahan et al. 2010; Carman et al. 2010; Sephton et al. 2011; Pyo et al. 2012); while a surprisingly low number of studies have explored the links between these harbors, including patterns of species turnover (beta-diversity), harbor type (recreational, fishing, commercial or mixtures thereof), or temporal or geographic trends (e.g. Lambert and Lambert 2003; Cohen et al. 2005; Grey 2009a).

The Mediterranean is the largest enclosed sea on Earth and is connected to most parts of the world by substantial maritime traffic (Kaluza et al. 2010; Keller et al. 2011), although vessels from the North Atlantic represent over 55 % of all entries (CIESM 2002). The shipping industry is largely responsible for the introduction of alien species from distant areas into the Mediterranean Sea and is one of the major vectors of spread, second only to corridors such as the Suez Canal (Zenetos et al. 2012). In addition, the highly urbanized Mediterranean Sea supports a dense network of harbors and marinas, especially along the northwestern coast (Airoldi and Beck 2007). Thus, the Mediterranean Sea is a well-suited location to test the importance of harbors as entrance gates to exotic species, while the densely packed northwestern coast and its high number of harbors and marinas allow testing relationships between species diversity, harbor type, and geographic distance to uncover patterns of secondary spread. Moreover, the enclosed nature of Mediterranean harbors allows for immediate quarantine and confined attempts of eradication should a known invader arrive. In this sense, knowledge of the processes of secondary spread can be used to define internal borders (Forrest et al. 2009), and direct contingency responses to maximize efficiency.

Ascidians or sea-squirts (Chordata, Tunicata) are sessile invertebrates ideally suited for the study of introduction processes as related to harbor dynamics. Firstly, ascidians are especially abundant on artificial substrates and are among the taxa with the highest recorded number of introduced species (Lambert and Lambert 1998, 2003; Paulay et al. 2002; Callahan et al. 2010; Aldred and Clare 2014). Secondly, ascidian larvae are short-lived and usually settle within a few hours or days (Svane and Young 1989; Ayre et al. 1997; Rius et al. 2010a, b) so these animals mostly rely on human transport for their long-distance dispersal. Furthermore, recurrent introductions are common in ascidians, increasing propagule pressure and, therefore, the probability of success of an introduction (Dupont et al. 2010; Goldstein et al. 2011; Pineda et al. 2011; Rius et al. 2012).

Successfully introduced ascidians have a series of biological characteristics that enable them to quickly become established in a new habitat, including the ability to outcompete resident species (Rius et al. 2009b) and high growth and reproductive outputs (Rius et al. 2009a; Morris and Carman 2012; Pineda et al. 2013). The long-term establishment of a non-indigenous ascidian also depends on both the physical (e.g., temperature, salinity) and biological (resident biota) conditions characterizing the new habitat (Brunetti et al. 1980; Vázquez and Young 2000; Whitlatch and Osman 2009; Bullard and Whitlatch 2009; Pineda et al. 2012a, b). To date there are few instances of introduced ascidians becoming invasive and spreading to natural habitats (Castilla et al. 2004; Turon et al. 2007; Rius et al. 2009b; Lambert 2009; Morris et al. 2009; Morris and Carman 2012; Stefaniak et al. 2009, 2012), but plenty of ascidians have established themselves on artificial substrates as fouling organisms, increasing management costs and impairing the normal development of commercial species in aquaculture facilities (reviewed in Aldred and Clare 2014).

The main aim of this study was to uncover patterns of secondary (post-border) spread of introduced benthic species in highly urbanized areas since some harbor types are known to be reservoirs for further spread while others act as sinks for migrants (Dupont et al. 2009). To achieve this goal, we performed a thorough inventory of the ascidian fauna in 32 Mediterranean harbors spanning the highly urbanized Catalan shores (NE Iberian Peninsula). These data were used to (1) characterize the presence and abundance of introduced species (2) analyze the role of harbors in the spread of introduced species by assessing patterns of diversity as a function of harbor type and geographic distance, and (3) establish a baseline for future studies.

## Materials and methods

### Sample collection

**Fig. 1:**
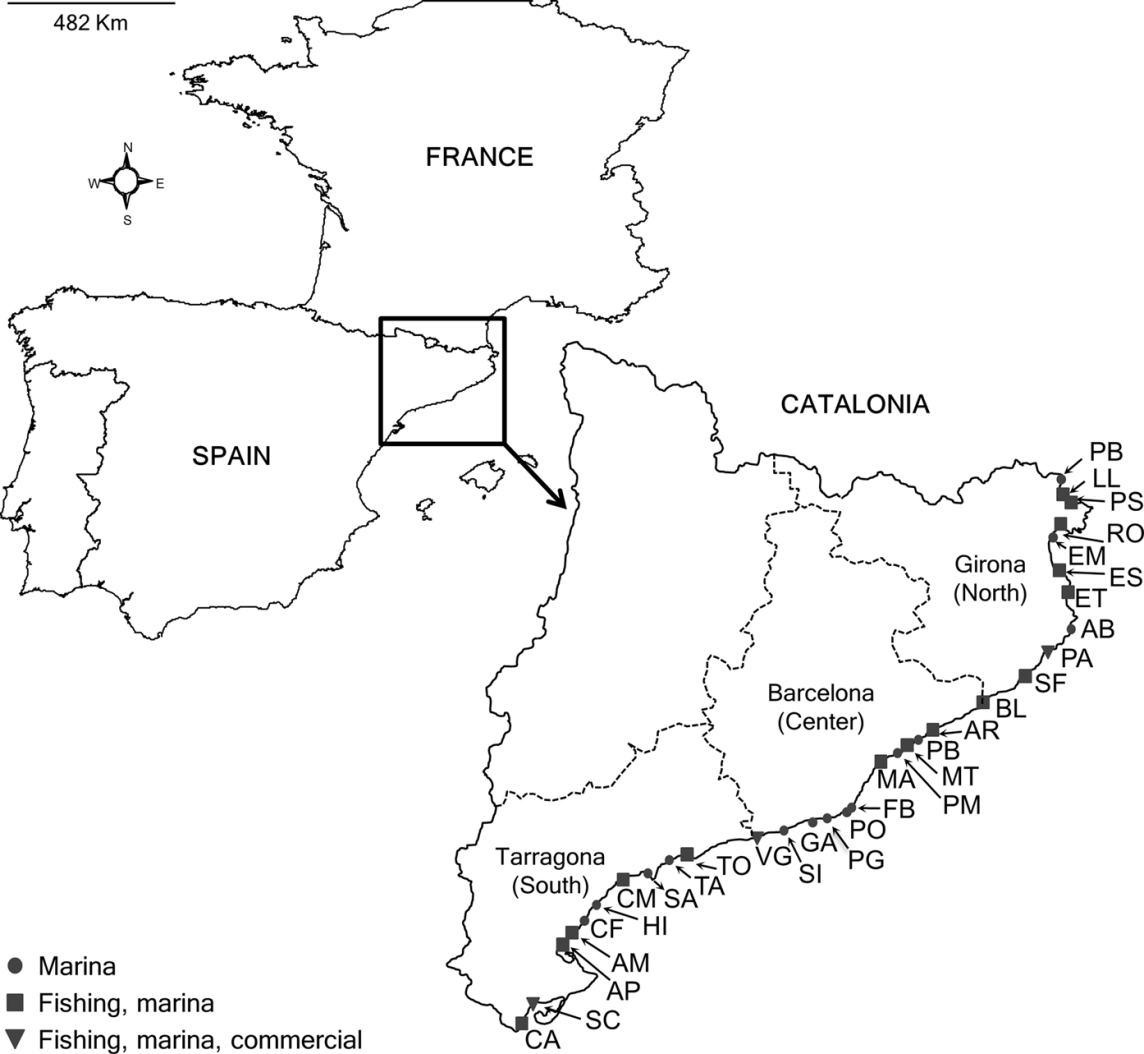
Map of the study area indicating the harbors surveyed (codes as in Table 1)

Thirty-two harbors along ca. 300 km of the Catalan coast (NE Iberian Peninsula) were surveyed between November 2012 and April 2013 (Fig. 1) and classified in three categories according to the type of activities observed (Table 1): (1) recreational marina, (2) marina and fishing, and (3) marina, fishing and commercial (vessels from local businesses; e.g. diving boats, tourist boats). Both fishing and commercial vessels in the investigated harbors operate daily and do not normally navigate overnight or internationally. The surveyed harbors provide a broad representation of small- to medium-sized harbors along the Western Mediterranean coast, ranging from 118 m (linear length) of concrete docks to 3,271 m (data obtained either from the harbor’s website or measured from aerial photographs using the software ImageJ; Table 1). The two largest commercial ports in Catalonia are located in the cities of Barcelona and Tarragona, and to date they are the only ones housing big cruise vessels, cargo ships, oil tankers and other vessels traveling internationally for trade. Unfortunately, these two ports could not be surveyed due to logistic reasons but their absence should not prevent us from observing patterns of secondary spread, since these are more likely to be dictated by the intense local traffic between medium and small harbors.

**Table 1:** List of the 32 harbors surveyed in this study, with name, code, type (1: marina, 2: marina and fishing, 3: marina, fishing and commercial), sampling date, geographic region (or province, north: Girona; central: Barcelona; south: Tarragona), GPS position, total linear length (meters) of all docks per harbor, and number of species observed at each harbor

Harbor name	Code	Type	Sampling date	Location	GPS position	Dock length	No. of species
St. Carles de la Ràpita	SC	3	November 9, 2012	South	40°34.50′N; 0°33.20′E	2,286	10
Port Balís	PL	1	December 31, 2012	Center	41°33.50′N; 2°30.50′E	2,327	8
Arenys de Mar	AR	2	January 19, 2013	Center	41°34.30′N; 2°33.30′E	2,314	9
Aiguablava	AB	1	January 29, 2013	North	41°56.00′N; 3°13.00′E	118	4
Estartit	ET	2	January 29, 2013	North	42°03.10′N; 3°12.40′E	1,100	7
L’Escala	ES	2	January 30, 2013	North	42°07.00′N; 3°08.60′E	2,923	16
Roses	RO	2	January 31, 2013	North	42°15.20′N; 3°10.60′E	2,780	11
Empuriabrava	EM	1	January 31, 2013	North	42°14.60′N; 3°08.10′E	557	5
Port de la Selva	PS	2	February 1, 2013	North	42°20.20′N; 3°11.90′E	1,041	11
Portbou	PB	1	February 2, 2013	North	42°25.70′N; 3°10.00′E	573	6
Blanes	BL	2	March 21, 2013	North	41°40.30′N; 2°47.80′E	1,486	8
Fòrum Barcelona	FB	1	March 22, 2013	Center	41°24.91′N; 2°13.72′E	1,485	9
Garraf	GA	1	March 1, 2013	Center	41°14.97′N; 1°54.04′E	956	6
Llançà	LL	2	February 1, 2013	North	42°22.00′N; 3°09.00′E	1,648	7
Masnou	MA	2	February 26, 2013	Center	41°28.50′N; 2°18.60′E	2,679	9
Mataró	MT	2	February 16, 2013	Center	41°32.00′N; 2°26.00′E	1,796	9
Port Ginesta	PG	1	March 12, 2013	Center	41°15.50′N; 1°55.50′E	3,271	7
Port Olímpic	PO	1	March 14, 2013	Center	41°23.12′N; 2°12.60′E	1,864	9
Premià de Mar	PM	1	March 25, 2013	Center	41°29.00′N; 2°21.00′E	1,132	10
Salou	SA	1	March 27, 2013	South	41°04.40′N; 1°07.80′E	502	8
Sant Feliu de Guíxols	SF	2	March 17, 2013	North	41°46.30′N; 3°01.54′E	1,202	7
Sitges (Aiguadolç)	SI	1	March 2, 2013	Center	41°13.90′N; 1°49.40′E	1,879	6
Port Nàutic Tarragona	TA	1	March 27, 2013	South	41°06.20′N; 1°15.80′E	955	9
Torredembarra	TO	2	January 28, 2013	South	41°08.03′N; 1°24.15′E	1,363	6
Vilanova i la Geltrú	VG	3	January 28, 2013	South	41°12.30′N; 1°43.70′E	3,012	6
Cambrils	CM	2	March 28, 2013	South	41°03.70′N; 1°03.80′E	1,482	9
Hospitalet de l’Infant	HI	1	March 28, 2013	South	40°59.23′N; 0°55.40′E	1,081	11
Calafat	CF	1	March 29, 2013	South	40°55.90′N; 0°51.20′E	754	5
Ametlla de Mar	AM	2	March 29, 2013	South	40°52.00′N; 0°47.00′E	1,477	8
Ampolla	AP	2	March 29, 2013	South	40°48.00′N; 0°43.00′E	1,809	10
Cases d’Alcanar	CA	2	March 30, 2013	South	40°33.20′N; 0°32.00′E	992	9
Palamós	PA	3	April 4, 2013	North	41°50.50′N; 3°07.10′E	1,549	7

Sampling was achieved using a variant of the Rapid Assessment Method described by Campbell et al. (2007) and consisted of monitoring at least 6 docks for each harbor (always including a central dock, an inner dock, and the dock located closest to the harbor entrance). When a marina (recreational activity) had more than 6 docks, then we monitored 6 docks plus one of every two (e.g. if a harbor had 12 docks, we monitored 9). All docks dedicated to fishing and commercial activities were sampled since there were not as numerous. Surveys were always conducted by the same 2–4 people (all well-trained in recognizing ascidian species) and lasted between 30 min (Aiguablava) to over 5 h (L’Escala), depending on the harbor size and type.

At each harbor, we recorded all ascidian species observed by pulling ropes, examining submerged structures, and turning around partially submerged buoys. Other substrata such as tires and plastic structures were occasionally observed hanging from docks and these were also monitored. Surveying organism diversity from the surface has been proven to be highly efficient (Grey 2009b) but, when possible, we also surveyed ship hulls and the harbor’s walls and bottom by submerging an underwater camera and observing the resulting digital pictures. Underwater photos were used with the sole purpose of verifying that all species have been sampled and to identify other substrata favored by ascidians since our goal was to maximize coverage and obtain exhaustive species lists from each sampled harbor. In addition, salinity was measured at −0.20 m with a refractometer.

At the end of each survey, relative abundance was estimated according to the number of individuals (or colonies) observed: (1) rare: one or a few specimens observed; (2) common: species frequently observed but not overly abundant; and (3) abundant: species occurring frequently and in great numbers. When the species was not readily recognized or we had any doubt about its taxonomic identification, we collected samples and preserved them in 4 % formaldehyde. Identification of preserved samples was based on standard taxonomic keys for ascidians and, particularly, on comprehensive faunistic studies of ascidians in the area (e.g., Turon 1987; Ramos-Esplá 1988).

### Ascidian barcoding

Most ascidian species retrieved in this study were barcoded using the standard 5′ “barcode region” of the mitochondrial gene cytochrome oxidase I (COI) to facilitate future identifications. Some rare species for which we only had material fixed in formaldehyde could not be sequenced (Table 2) and we choose not to sequence *Styela plicata* because hundreds of sequences have been recently obtained for this species in the same study area (Pineda et al. 2011). For each of the other species, at least one individual or colony was immediately preserved in absolute ethanol and stored at −20 °C until processed. To maximize DNA extractions, specimens were dissected under a stereomicroscope to separate zooids from the tunic for colonial species and a piece of the branchial sac for solitary ones. DNA was extracted from the zooid fraction or the branchial sac tissue using the DNeasy Blood and Tissue kit (Qiagen). A ca. 600 bp fragment of the COI gene was amplified using either the primer set Tun_forward and Tun_reverse2 described by Stefaniak et al. (2009) or the primer set LCO1490 and HCO2198 described by Folmer et al. (1994). Total PCR volume was 25 µl, including 5 pmol of each primer, 5 nmol of each dNTP, 1× reaction buffer (Ecogen), and 2.5 units of BIOTAQ polymerase (Ecogen). Amplification conditions included initial DNA denaturing at 94 °C for 5 min, followed by 40 amplification cycles of 94 °C for 30 s, annealing at 40 °C for 30 s, and extension at 72 °C for 1 min, and a final extension step at 72 °C for 10 min. PCR cleaning and sequencing reactions were performed at Macrogen, Inc. (Seoul, Korea). The resulting 136 sequences were deposited in the GenBank database (accession numbers KF309529–KF309664).

**Table 2:** Ascidian species (classified at least to the genus level) found in the 32 surveyed harbors

Order	Species	Origin	Acc. num.	# Harbors
Aplousobranchia	*Clavelina oblonga*	Introduced	KF309648	1
	*Clavelina lepadiformis*	Introduced	KF309563, -638	32
	*Clavelina sabbadini*	Native	KF309535, -645	2
	*Diplosoma listerianum*	Introduced	KF309531, -561, -581, -605, -616, -638, -639, -660, -664	30
	*Diplosoma spongiforme*	Native	KF309624	2
	*Trididemnum cereum*	Native	KF309632	3
	*Didemnum* sp. 1	–	KF309573	1
	*Didemnum* sp. 2	–	KF309622	1
	*Didemnum fulgens*	Native	KF309576	1
	*Morchellium argus*	Native	KF309620-21	1
	*Aplidium accarense*	Introduced	KF309553, -558, -571, -574, -584–586, -597–599, -601, -646, -618, -625–27, -629–630, -640, -654–657, -663	23
	*Aplidium* sp. 1	–	–	1
	*Aplidium* sp. 2	–	KF309633	1
Phlebobranchia	*Ascidia virginea*	Native	KF309647	1
	*Ascidia* sp.	–	–	1
	*Ascidiella aspersa*	Introduced	KF309533-34, -555, -559, -562, -568, -594, -606, -617, -631, -637, -653, -661	27
	*Ascidiella scabra*	Cryptogenic	KF309529, -556, -560, -572, -650	5
	*Phallusia ingeria*	Native	–	1
	*Phallusia* sp.	–	–	1
	*Phallusia mammillata*	Native	KF309607	1
	*Phallusia fumigata*	Native	KF309548	1
	*Ciona intestinalis*	Cryptogenic	KF309614, -532, -554, -570, -574, -578, -580, -587, -591, -593, -602–604, -613, -628, -651, -658, -662	28
	*Ciona* sp.	–	KF309636	1
Stolidobranchia	*Botrylloides leachii*	Cryptogenic	KF309549, -551, -608–611, -641–642, -644	3
	*Botryllus schlosseri*	Cryptogenic	KF309536-47, -530, -564–567, -579, -592, -615, -659	30
	*Polyandrocarpa zorritensis*	Introduced	KF309643	1
	*Distomus variolosus*	Native	KF309623	2
	*Styela plicata*	Introduced	–	21
	*Styela canopus*	Cryptogenic	KF309590	2
	*Polycarpa pomaria*	Native	–	2
	*Polycarpa* sp.	–	KF309588	1
	*Molgula bleizi*	Native	–	2
	*Molgula occidentalis*	Native	–	1
	*Molgula* sp.	–	–	1
	*Microcosmus squamiger*	Introduced	KF309550, -552, -595, -600, -612, -619, -634–635, -649, -652	24
	*Pyura dura*	Native	KF309596	4
	*Pyura squamulosa*	Native	–	2

Species were further classified according to their origin: native, introduced, and cryptogenic (see Appendix 1). GenBank accession numbers of the COI sequences generated in this study and the number of harbors in which the species have been found are also indicated

### Data analysis

Once identified, each species was classified as native, introduced, or cryptogenic (Carlton 1996), following relevant literature (see Appendix 1). In short, introduced species were those for which distributional or genetic data existed supporting an alien origin. Native species were those endemic to the Mediterranean, or with Atlanto-Mediterranean distribution, known to inhabit natural substrata not adjacent to artificial structures. Finally, cryptogenic species were those for which there was insufficient information to unambiguously decide whether they were introduced or native. The species classified as cryptogenic were widely distributed and generally abundant within harbors, so an introduced origin was suspected in most cases. However, since no study to date has determined their native area and in order to avoid errors, cryptogenic species were not included in further analyses comparing introduced and native species.

A linear regression analysis was performed to determine whether there was a relationship between harbor size (as linear length of docks) and number of species recorded. Analyses of variance (ANOVA) were used to test for differences in linear length among harbor types or geographic zones. Likewise, tests were done to compare species abundance across harbor types and geographic zones. When the assumptions of normality or homogeneity of variances were not met, the non-parametric Kruskal–Wallis test was used instead of ANOVA. All tests were performed using the software SigmaStat v 3.5. To compare ascidian diversity and structure across harbors two similarity matrices were constructed. The first considered the relative abundance of each species within each harbor using the Bray-Curtis index (no transformation of data was applied, as our original data was semi-quantitative). The second dataset consisted of presence–absence data analyzed with the Jaccard index. Analyses were carried out using the Primer v6.1.10 statistical package (Clarke and Gorley 2006) with the PERMANOVA + β20 module (Anderson et al. 2008) incorporated. Permutational analyses of variance (PERMANOVA) were applied to assess the significance of the factors: geographic location (North, Center, South, which corresponded to the three provinces in the area: Girona, Barcelona, and Tarragona, respectively) and harbor type (three levels, see above). In the case of significant factors, we ran permutational pairwise tests on levels of these factors. Likewise, we analyzed differences in relative dispersion among the groups determined by levels of significant factors using PERMDISP. This was done to verify that the significant outcome in PERMANOVA was due to differences in location in multivariate space, not to differences in dispersion among the groups. These analyses were performed for three datasets, one comprising all species (32 harbors), one with only introduced species (32 harbors), and one with just the species identified as native (a restricted set of the 15 harbors were native species were found).

Results were visualized with non-metric multidimensional scaling (nMDS) plots. These analyses were done with R v 2.14.2 (R Development Core Team 2012). The similarity matrices were transformed to distances for input into the vegan 2.0-7 package (Oksanen et al. 2013). We used the metaMDS function of vegan with default parameters. Unlike MDS programs that find a single configuration by iteration and thus can get trapped in local optima, metaMDS performs different (20) random starts and compares them to find a stable solution. MDS configurations were obtained separately for the entire dataset comprising all species, for the introduced species, and for the native species. The analyses were also run separately for relative abundance and presence–absence data and compared using Procrustes analysis (Peres-Neto and Jackson 2001) as implemented in vegan (function Protest), and the significance of the correlations found was tested by permutation. For the native species dataset, the final solutions reached were not stable among runs due to the low number of data, even after increasing the number of random starts (parameter trymax) to one hundred. For this reason MDS plots for the native species are not shown.

Additionally, Mantel tests were conducted to test for correlations between geographic distances among harbors (in kilometers) and species dissimilarity for both the abundance and the presence–absence data involving all species, introduced species, and native species. Shortest surface distances between pairs of harbors were calculated using free software developed by Byers (1997). The Mantel tests were performed using the ade4 package for R (function mantel.rtest) and its significance tested by permutation (Dray and Dufour 2007).

## Results

### Ascidian diversity

In the 32 harbors investigated, we identified 28 ascidians at the species level and another 9 at the genus level (Table 2; Fig. S1). Samples that could not be assigned a species name were normally single specimens at immature stages, so key morphological characters could not be observed (and, for those barcoded, no perfect match was found in the databases either). Consequently, these taxa could not be further classified according to their origin and were only used in analyses using the global dataset. On average, Catalan harbors contained 8.18 ± 2.33 (SD) ascidian species. The harbor that presented the highest species richness was l’Escala (*n* = 16), followed by Roses, Port de la Selva and Hospitalet de l’Infant, each with 11 species (Table 1). In contrast, we only found 5 species in Empuriabrava and Calafat, and 4 in Aiguablava (Table 1). A significant relationship between harbor size and species richness was observed (linear regression, *p* < 0.05), with harbor size explaining 17.2 % of observed variance in species richness among harbors (Fig. S2). However, no significant differences in size were observed between harbor types (ANOVA, *F* = 3.060, DF = 2,29, *p* = 0.062) or between geographic zones (*F* = 1.928, DF = 2,29, *p* = 0.164). The data on species and abundances found at each harbor are listed in Table S1. Most species were either known introduced ascidians (*n* = 8) or were considered native from the area (*n* = 15), while 5 were assigned cryptogenic status (Table 2; Appendix 1). All species found had been previously reported in the Mediterranean Sea, with the exception of *Aplidium accarense* (see further taxonomic remarks in Appendix 2).

**Fig. 2:**
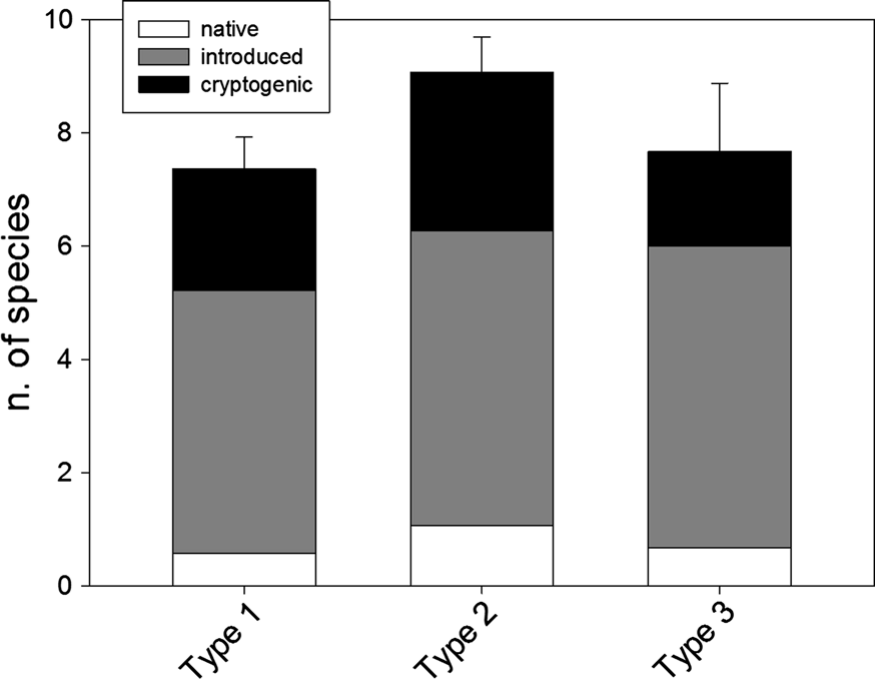
Mean number of ascidian species found at each harbor type (type 1: marina; type 2: marina and fishing; type 3: marina, fishing and commercial) and for each category of species. *Bars* are standard errors

**Fig. 3:**
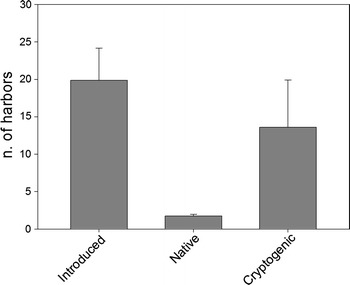
Mean number of harbors in which each species was found as per type of species (introduced, native, cryptogenic). *Bars* are standard errors

No significant differences in species number were found according to harbor type (Fig. 2, ANOVA *F* = 2.179, DF = 2,29, *p* = 0.131) or geographical area (*F* = 0.016, DF = 2,29, *p* = 0.984). Introduced and cryptogenic species were the most common in all harbor types (Fig. 2). The geographic span of the three groups of species (introduced, cryptogenic and native) was clearly different (Fig. 3). Introduced species were present in many more harbors (an average ca. 20 harbors) than native species (average of 1.7 harbors), while cryptogenic species were found in 13.6 harbors on average. The differences in spread between native and the other two groups of species were significant, but not between introduced and cryptogenic forms (Kruskal–Wallis test, *H* = 10.371, DF = 2, *p* = 0.006, followed by Dunn’s pairwise tests at *p* = 0.05).

Both colonial and solitary ascidians were widely distributed among harbors (Table 2). The ascidian *Clavelina lepadiformis* was found in all 32 sampled harbors, while *Botryllus schlosseri* and *Diplosoma listerianum* were observed in 30. The most common solitary ascidian was *Ciona intestinalis* (present in 28 harbors) followed by *Ascidiella aspersa* (27 harbors) and *Microcosmus squamiger* (24 harbors). All these species were classified as introduced or cryptogenic and were observed colonizing a wide range of substrata, including ropes, buoys, tires, boat hulls and metal ladders. On the other hand, the least common ascidians (identified at the species level) were the colonial forms *Clavelina oblonga, Morchellium argus, Didemnum fulgens*, and *Polyandrocarpa zorritensis*; and the solitary species *Molgula occidentalis*, *Ascidia virginea*, *Phallusia ingeria*, *P. mammillata* and *P. fumigata*. These species were observed in a single harbor and were exclusively found on ropes; except for *P. ingeria* and *D. fulgens* that grew in mussels and *P. zorritensis* that was also observed under buoys and attached to dock walls.

Twenty-seven species were identified genetically (Table 2). The sequences generated for *Clavelina lepadiformis* corresponded to the introduced Atlantic clade defined in Turon et al. (2003). All sequences obtained for *Diplosoma listerianum* corresponded to clade A (Pérez-Portela et al. 2013), sequences for *C. intestinalis* matched species A described in Caputi et al. (2007) and Nydam and Harrison (2007), and sequences for *B. schlosseri* corresponded to clade 5 in López-Legentil et al. (2006), except for KF309545 that matched clade 1, and two sequences (KF309592, KF309530) that presented 98 % identity (BLASTn) with a USA specimen (GU065352, Callahan et al. 2010). Identification of *Ascidiella scabra* and *A. aspersa* was made based on morphological characters following a recent review (Nishikawa et al. 2014). However, while the COI sequences generated for *A. aspersa* clustered within the *A. aspersa* clade of Nishikawa et al. (2014), those of *A. scabra* formed a well-supported clade separated from the corresponding clade in that work, which only included European Atlantic specimens (Fig. S3).

Permutational analyses considering both geographic location and harbor type as variables showed that there was a significant effect of both factors on ascidian community structure, which together explained about 28–30 % of the variation found among harbors (Table 3). No significant interaction between these two variables was found, indicating that the effect of each variable on our data was independent of the other. The results were similar when considering relative abundance and presence–absence data. The PERMDISP analyses were not significant (Table 3), indicating homogeneity in data dispersion across levels of the considered factors, except for the geographic region factor with the presence–absence data matrix (*p* = 0.036). When the analyses were run separately for introduced and native species, the same results were obtained for the introduced species dataset (Table 3), and in this case no evidence for heterogeneity of dispersion between groups was found (non-significant PERMDISP outcome). The native species distribution did not show any significant pattern according to harbor type or geographic location.

**Table 3:** Permutational statistical analyses (PERMANOVA) of ascidian similarity among harbors according to their type (marina; marina and fishing, and marina, fishing and commercial) and geographic region (north: Girona; central: Barcelona; south: Tarragona)

	*df*	Abundance				Presence–absence			
		*SS*	*pseudo*-*F*	*p * value	*Permdisp*	*SS*	*pseudo*-*F*	*p * value	*Permdisp*
All species									
Region	2	3,685.5	2.578	0.006	0.128	4,408	2.462	0.009	0.036
Harbor type	2	3,252.4	2.275	0.020	0.366	3,484.6	1.946	0.038	0.714
Interaction	4	3,384.8	1.184	0.287	–	4,879.6	1.363	0.138	–
Residual	23	16,440				20,588			
Introduced species									
Region	2	3,759.8	3.306	0.003	0.308	3,871.8	4.513	0.004	0.249
Harbor type	2	2,513.2	2.210	0.042	0.190	3,871.8	4.513	0.004	0.249
Interaction	4	2,597.4	1.142	0.375	–	3,171.5	1.848	0.095	–
Residual	23	13,079				9,866.4			
Native species									
Region	2	10,382	1.238	0.227	–	10,057	1.153	0.318	–
Harbor type	2	9,128.7	1.089	0.356	–	9,376.1	1.076	0.361	–
Interaction	2	9,485.8	1.132	0.347	–	9,390.6	1.077	0.378	–
Residual	8	33,533				34,867			

Analyses were performed for abundance (Bray-Crutis index) and presence–absence (Jaccard index) data, and for the global dataset (32 harbors), the introduced species dataset (32 harbors), and the native species dataset (15 harbors). PERMISP probabilities of homogeneity of dispersion were also given for significant factors

Pairwise comparisons of levels of the significant main factors revealed that, for the geographic regions, there were significant differences between the North and the other two zones (Center and South), which were not different among themselves (Table 4). For the presence–absence dataset, the PERMDISP analyses also showed a significantly higher dispersion of the data in the North and South than in the Center. Pairwise comparisons between harbor types revealed significant differences in the comparison between marinas (type 1) and marina and fishing harbors (type 2) when considering relative abundance values for both the total species and the introduced species datasets (Table 5). Results were less clear-cut for the presence–absence data, as no comparison was significant when considering all species, and only the comparison between harbor types 2 and 3 (marina, fishing and commercial) was significant for the introduced species (Table 5).

**Table 4:** Permutational pairwise comparisons of ascidian similarity among harbors according to geographic zone (north: Girona; central: Barcelona; south: Tarragona) for all species and for the introduced species dataset

	Abundance		Presence–absence	
	*t*	*p * value	*t*	*p * value
All species				
South–Center	1.283	0.180	1.471	0.091
South–North	1.599	0.024	1.531	0.025
Center–North	1.810	0.017	1.681	0.018
Introduced species				
South–Center	1.078	0.360	1.265	0.229
South–North	1.925	0.011	2.264	0.006
Center–North	2.118	0.010	2.401	0.010

**Table 5:** Permutational pairwise comparisons of ascidian similarity among harbors according to their type (type 1: marina; type 2: marina and fishing; type 3: marina, fishing and commercial) for all species and for the introduced species dataset

	Abundance		Presence–absence	
	*t*	*p * value	*t*	*p * value
All species				
Type 1–type 2	1.585	0.027	1.365	0.066
Type 1–type 3	1.443	0.118	1.412	0.102
Type 2–type 3	1.452	0.074	1.424	0.090
Introduced species				
Type 1–type 2	1.711	0.042	1.664	0.091
Type 1–type 3	1.345	0.168	1.584	0.121
Type 2–type 3	1.149	0.293	1.992	0.038

**Fig. 4:**
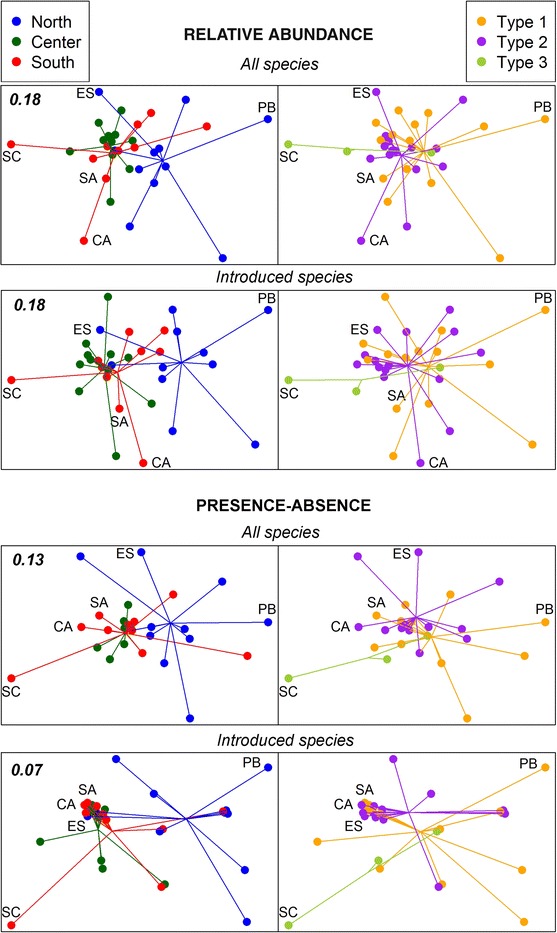
Non-metric MDS plots of the harbors studied obtained from the relative abundance and the presence–absence data, for the whole dataset and for the introduced species. Every plot is color-coded for the geographic region (*left; north* Girona; *central* Barcelona; *south* Tarragona) and for the type of harbor (*right; type 1* marina; *type 2* marina and fishing; *type 3* marina, fishing and commercial). Lines join harbors with their weighed group centroid as for the corresponding factor. Coincident positions of the presence–absence plots were slightly displaced as overlapping groups for clarity. Some harbors are identified (codes as in Table 1) to ease comparison of the configurations. MDS plots with full code names are given in Fig. S4. Stress values are given for each plot (*upper left*)

Non-metric MDS plots constructed from relative abundance data (using Bray–Curtis index) showed better differentiation among harbors than those based on presence–absence data (Jaccard index, Fig. 4). The differences observed in PERMANOVA analyses according to geographic location are graphically represented by a separation of the group centroids; the northern harbors in particular tended to be separated from the center and southern harbors, which clustered more closely (Fig. 4). Differences according to harbor type were less evident, with all group centroids relatively close together. Some type 1 harbors appeared consistently separated from the other harbors, while a type 3 harbor (SC: Sant Carles) was usually set apart at one extreme of the spatial ordination (Fig. 4), due to the presence of some particular species at this harbor that were not found in other harbors (see Discussion). Overall, the spatial harbor arrangement was coherent between the MDS plots of the whole datasets and those of introduced species and, to a lesser degree, between abundance and presence–absence data (Fig. 4). This was further corroborated by the results of the Procrustes analyses (Table 6), which showed high correlations (*r* > 0.84) between the whole species and the introduced species configurations (*p* < 0.001), and lower (*r* > 0.56), although significant (*p* < 0.001), correlations between the abundance and the presence–absence configurations (Table 6).

**Table 6:** Statistical comparison of the MDS configurations obtained. Procrustes sum of squares, correlation, and *p* value obtained by permutation are given

	Sum of squares	Correlation	*p* value
Abundance versus presence–absence			
All species	0.634	0.603	<0.001
Introduced species	0.656	0.586	<0.001
All species versus introduced species			
Abundance	0.124	0.936	<0.001
Presence–absence	0.293	0.841	<0.001

### Ascidian distribution along the Catalan coast

The shortest surface distance between the northernmost (Portbou) and southernmost (Cases d’Alcanar) harbors was 299.49 km, and between the closest harbors (Garraf and Port Ginesta) 2.26 km. A Mantel test showed a significant correlation between geographic distance and species dissimilarity (*r* = 0.321 for the relative abundance matrix, *r* = 0.325 for the presence–absence matrix, *p* < 0.001 in both cases). Similar results were obtained when correlating geographic distances with dissimilarity based only on introduced species (Mantel test, *r* = 0.271 for the relative abundance matrix, *r* = 0.270 for the presence–absence matrix, *p* < 0.001 in both cases). For the harbors with native species, the correlations between geographic distances and distances based on diversity of native ascidians were weaker and non significant (relative abundance data, *r* = 0.166, *p* = 0.080; presence–absence data, *r* = 0.165, *p* = 0.062).

## Discussion

This study uncovered an unexpected diversity of ascidians in northwestern Mediterranean harbors. Our survey of 32 small- to medium-sized harbors identified 28 ascidians at the species level and another 9 at the genus level. A recent review placed the total number of ascidian species in the Mediterranean at 229 (Coll et al. 2010), thus in just a single type of habitat along ca. 300 km of shoreline, we have found ca. 16 % of the total recorded biodiversity in the whole Mediterranean Sea. We also found a clear pattern of ascidian distribution, in which introduced (and cryptogenic) species tended to be present in many more harbors than native species, while native species abundance was low overall. This pattern reinforces the general understanding that harbors are not good habitats for native species and are instead populated by highly tolerant introduced forms. Thus, harbor connectivity through shipping does not contribute to the spread of indigenous species, but rather harbors and marinas are strongholds for dispersion of introduced forms. In addition, we found a significant and positive relationship between harbor size and species richness, indicating that larger harbors tended to contain more ascidian species. We did not observe, however, significant differences in the number of ascidian species according to harbor type or geographic zone.

A significant correlation between geographic distance and ascidian diversity in the harbors studied was detected. This correlation was mostly due to the distribution of introduced species, and was weaker and not significant when native species were considered. This pattern is likely a result of the short-range movement of vessels among the small- to medium-sized harbors that enable species dispersal, while species establishment is facilitated by environmental similarity between close-by harbors. It is noteworthy that, even in habitats subject to anthropogenic transport (which usually “breaks” isolation by distance patterns), differences can still be retrieved at the scale considered here (i.e. 300 km). This observation has important implications for secondary spread of introduced species and points to stepping-stone processes that are highly relevant for future preventive actions (see below).

Permutational analyses of variance revealed that harbor type and latitudinal position had significant effects on ascidian community structure, with patterns driven by the introduced species (both factors were not significant for native species). For the factor ‘harbor type’, pairwise tests showed significant (or marginally so) differences for many comparisons in one or another analysis (considering all species and introduced species, as well as relative abundance and presence–absence data), a fact likely reflecting the different size, boating dynamics, and maintenance levels of the different harbor types. Alternatively, the different number of harbors scored in each category (e.g., only three in category 3) may have also influenced some of the *p* values obtained.

For the factor ‘geographic location’, pairwise comparisons consistently showed a different composition between harbors located in the North (Girona), and the central and southern zones (Barcelona and Tarragona, respectively). Seawater temperatures in southern Catalonia are 0.1–2 °C warmer than in the North, depending on the season and year (López-Legentil et al. 2005; Sabatés et al. 2006). Some of the ascidian species found here are known to be very sensitive to changes in seawater temperature and feature resistance forms during summer (e.g. *Didemnum fulgens*, López-Legentil et al. 2013), while others such as *Styela plicata* are able to thrive in habitats featuring seasonal temperature variations of 23 °C (Pineda et al. 2012b). Thus, the absence of some species in northern or southern Catalonia could be due to differences in seawater temperatures, as found for other ascidian species (Lambert 2005). Alternatively, species that are present in just southern or northern harbors could be recent introductions that have not yet spread to harbors located further away.

In spite of a significant effect of the factors analyzed, together they explain ca. 30 % of the variability found, so other abiotic (e.g. salinity, pollution) and biotic factors are influencing ascidian populations. Salinity appears to be an important factor in determining the distribution of some introduced species (Dybern 1969; Lowe 2002; Epelbaum et al. 2009; Pineda et al. 2012a). Our salinity measurements were taken at one point in time and under different weather conditions and thus can only be considered ‘snapshots.’ Not surprisingly, we did not find any correlation between number of species and salinity values (*r* ^2^ = 0.001; results not shown). A potential exception was observed for the northern section of the harbor of Sant Carles de la Rapita that received a freshwater rivulet and for which we recorded a salinity of 15 ‰, although it is likely that salinity drops are episodic there. Notably, this section of the harbor was very different from the rest (34 ‰) and was colonized by only two species (out of a total of 10 recorded in that harbor): *Clavelina oblonga* and *Styela plicata*, both known to tolerate salinities <34 ‰ (Rocha et al. 2009; Pineda et al. 2012b).

Pollution is also known to shape benthic communities in harbors, especially heavy metals (Piola and Johnston 2009). Information about pollution levels at the investigated Catalan harbors is scarce, but the few studies conducted so far had reported moderate to low levels of heavy metals (De Caralt et al. 2002; Cebrian et al. 2007). It is known that some ascidian species such as *Ciona intestinalis, Microcosmus squamiger, Styela plicata, Diplosoma listerianum, Botrylloides leachii* and *Botryllus schlosseri* are able to tolerate high levels of pollution and that this tolerance has been key for their successful introduction in new habitats (Naranjo et al. 1996; Lambert and Lambert 2003; Piola and Johnston 2008; Pineda et al. 2012a). Finally, biotic factors such as competition and predation (Whitlatch and Osman 2009; Ordóñez et al. 2013) are also likely to have an impact on overall species abundance and distribution between and within harbors and their importance remains to be tested.

In general, relative abundance data (here given as semi-quantitative ranks) and presence–absence data tended to give similar information in the analyses performed. However, the ordination configurations with presence–absence data were less resolved and tended to clutter harbors, accentuating the importance of acquiring abundance data whenever possible. MDS plots showed that the overall ordination of harbors was largely driven by the distribution pattern of introduced species. Some northern harbors tended to appear separated from the rest, with the harbor from Sant Carles de la Ràpita (SC) offset from the others in ordination space. This separation was explained by the presence of some exclusive (*Polyandrocarpa zorritensis, Clavelina oblonga*) or almost exclusive (*Clavelina sabbadini*, *Botrylloides leachii*) species in this harbor. SC harbor is the home base of fishermen working in the nearby aquaculture facilities of the Ebro Delta, a large artificial setup hosting several introduced species (Ordóñez 2013). Thus, at least some of the species retrieved in SC may have come from the nearby aquaculture settings, and SC (which is large and sustains recreational activities, a considerable fishing fleet and some commercial ships) may now be acting as a focal point for further expansions. This observation revealed a complex interplay among harbor types, aquaculture activities in the vicinity, and secondary spread of introduced species.

In conclusion, we have uncovered an unexpected diversity of ascidian species in a relatively restricted but vastly developed stretch of coast in the Western Mediterranean. We also found an effect of harbor type, size and geographic area on ascidian diversity and distribution, as well as a pattern of higher similarity in geographically closer harbors. Thus, highly urbanized coastlines and their associated network of harbors and marinas act as dispersal strongholds for introduced species with little impact for the rarely observed native ascidians. Cataloguing species and establishing periodic surveys of artificial structures are easily achieved first-steps to prevent spreading of detrimental species and are critical for the development of cost-effective management and contingency plans. Species inventories should not only incorporate taxonomic surveys (for which complementary genetic data is mandatory in the face of taxonomic conundrums), but also a thorough assessment of inter-harbor distribution patterns in order to define efficient internal borders for further action.

## Electronic supplementary material

Below is the link to the electronic supplementary material.

Supplementary material 1 (DOCX 1853 kb)
