# Supplementary material for: Harbor networks as introduction gateways: contrasting distribution patterns of native and introduced ascidians
Source: Biol Invasions. 2014 Dec 10;17(6):1623–38. doi: 10.1007/s10530-014-0821-z (PMC4498637; doi:10.1007/s10530-014-0821-z)
Supplement: Supplementary file 1 — Supplementary material 1 (DOCX 1853 kb) [file 10530_2014_821_MOESM1_ESM.docx]

**SUPPLEMENTARY MATERIAL**

Biological Invasions

**Harbor as introduction gateways: contrasting distribution patterns of native and introduced ascidians**

Susanna López-Legentil^1,2,^* · Miquel L. Legentil^2^ · Patrick M. Erwin^1^ · Xavier Turon^3^

^1^*Department of Biology & Marine Biology and Center for Marine Science, University of North Carolina Wilmington, 5600 Marvin K. Moss Lane, 28409 Wilmington, United States of America*

*^2^Departament de Biologia Animal and Institut de Recerca de la Biodiversitat (IRBio), Universitat de Barcelona (UB), Diagonal Avenue 643, 08028 Barcelona, Spain*

^3^Center for Advanced Studies of Blanes (CEAB-CSIC), Accés Cala S. Francesc 14, 17300 Blanes, Girona, Spain

*Corresponding author: Susanna López-Legentil; email: [lopezlegentils@uncw.edu](mailto:lopezlegentils@uncw.edu)

**Table S1.** Abundance (semi-quantitative index, see main text) of the ascidian species found in the 32 harbors surveyed. Harbor codes as in Table 1.

| **Species** | **SC** | **PB** | **AR** | **AB** | **ET** | **ES** | **RO** | **EM** | **PS** | **PB** | **BL** | **FB** | **GA** | **LL** | **MA** | **MT** | **PG** | **PO** | **PM** | **SA** | **SF** | **SI** | **TA** | **TO** | **VG** | **CM** | **HI** | **CF** | **AM** | **AP** | **CA** | **PA** |
| --- | --- | --- | --- | --- | --- | --- | --- | --- | --- | --- | --- | --- | --- | --- | --- | --- | --- | --- | --- | --- | --- | --- | --- | --- | --- | --- | --- | --- | --- | --- | --- | --- |
| *Clavelina oblonga* | 2 | 0 | 0 | 0 | 0 | 0 | 0 | 0 | 0 | 0 | 0 | 0 | 0 | 0 | 0 | 0 | 0 | 0 | 0 | 0 | 0 | 0 | 0 | 0 | 0 | 0 | 0 | 0 | 0 | 0 | 0 | 0 |
| *Clavelina lepadiformis* | 3 | 2 | 3 | 2 | 3 | 1 | 2 | 2 | 2 | 1 | 3 | 3 | 1 | 2 | 3 | 3 | 3 | 3 | 3 | 3 | 3 | 1 | 1 | 3 | 2 | 3 | 3 | 3 | 3 | 3 | 1 | 3 |
| *Clavelina sabbadini* | 1 | 0 | 1 | 0 | 0 | 0 | 0 | 0 | 0 | 0 | 0 | 0 | 0 | 0 | 0 | 0 | 0 | 0 | 0 | 0 | 0 | 0 | 0 | 0 | 0 | 0 | 0 | 0 | 0 | 0 | 0 | 0 |
| *Diplosoma listerianum* | 0 | 1 | 3 | 1 | 3 | 3 | 2 | 2 | 3 | 0 | 3 | 2 | 2 | 2 | 3 | 3 | 3 | 2 | 3 | 2 | 2 | 3 | 3 | 3 | 2 | 3 | 2 | 3 | 3 | 2 | 1 | 2 |
| *Diplosoma spongiforme* | 0 | 0 | 0 | 0 | 0 | 0 | 0 | 0 | 1 | 1 | 0 | 0 | 0 | 0 | 0 | 0 | 0 | 0 | 0 | 0 | 0 | 0 | 0 | 0 | 0 | 0 | 0 | 0 | 0 | 0 | 0 | 0 |
| *Trididemnum cereum* | 0 | 0 | 0 | 0 | 0 | 1 | 1 | 0 | 0 | 0 | 0 | 0 | 0 | 0 | 0 | 0 | 0 | 0 | 1 | 0 | 0 | 0 | 0 | 0 | 0 | 0 | 0 | 0 | 0 | 0 | 0 | 0 |
| *Didemnum* sp. 1 | 0 | 0 | 0 | 0 | 0 | 1 | 0 | 0 | 0 | 0 | 0 | 0 | 0 | 0 | 0 | 0 | 0 | 0 | 0 | 0 | 0 | 0 | 0 | 0 | 0 | 0 | 0 | 0 | 0 | 0 | 0 | 0 |
| *Didemnum* sp. 2 | 0 | 0 | 0 | 0 | 0 | 0 | 0 | 0 | 1 | 0 | 0 | 0 | 0 | 0 | 0 | 0 | 0 | 0 | 0 | 0 | 0 | 0 | 0 | 0 | 0 | 0 | 0 | 0 | 0 | 0 | 0 | 0 |
| *Didemnum fulgens* | 0 | 0 | 0 | 0 | 0 | 1 | 0 | 0 | 0 | 0 | 0 | 0 | 0 | 0 | 0 | 0 | 0 | 0 | 0 | 0 | 0 | 0 | 0 | 0 | 0 | 0 | 0 | 0 | 0 | 0 | 0 | 0 |
| *Morchellium argus* | 0 | 0 | 0 | 0 | 0 | 0 | 0 | 0 | 1 | 0 | 0 | 0 | 0 | 0 | 0 | 0 | 0 | 0 | 0 | 0 | 0 | 0 | 0 | 0 | 0 | 0 | 0 | 0 | 0 | 0 | 0 | 0 |
| *Aplidium accarense* | 2 | 2 | 3 | 0 | 0 | 3 | 0 | 0 | 0 | 0 | 2 | 3 | 2 | 0 | 3 | 2 | 3 | 2 | 3 | 1 | 0 | 3 | 1 | 2 | 3 | 3 | 2 | 0 | 2 | 2 | 1 | 1 |
| *Aplidium* sp. 1 | 0 | 0 | 0 | 0 | 0 | 1 | 0 | 0 | 0 | 0 | 0 | 0 | 0 | 0 | 0 | 0 | 0 | 0 | 0 | 0 | 0 | 0 | 0 | 0 | 0 | 0 | 0 | 0 | 0 | 0 | 0 | 0 |
| *Aplidium* sp. 2 | 0 | 0 | 0 | 0 | 0 | 0 | 0 | 0 | 0 | 0 | 0 | 0 | 0 | 0 | 0 | 0 | 0 | 0 | 3 | 0 | 0 | 0 | 0 | 0 | 0 | 0 | 0 | 0 | 0 | 0 | 0 | 0 |
| *Botrylloides leachii* | 3 | 0 | 0 | 0 | 0 | 0 | 0 | 0 | 0 | 0 | 0 | 0 | 0 | 0 | 0 | 0 | 0 | 0 | 0 | 0 | 0 | 0 | 0 | 0 | 0 | 0 | 0 | 0 | 0 | 2 | 1 | 0 |
| *Botryllus schlosseri* | 2 | 3 | 3 | 1 | 3 | 3 | 2 | 3 | 1 | 0 | 3 | 3 | 2 | 2 | 3 | 3 | 2 | 2 | 3 | 2 | 2 | 2 | 1 | 2 | 1 | 3 | 2 | 0 | 2 | 2 | 1 | 2 |
| *Polyandrocarpa zorritensis* | 3 | 0 | 0 | 0 | 0 | 0 | 0 | 0 | 0 | 0 | 0 | 0 | 0 | 0 | 0 | 0 | 0 | 0 | 0 | 0 | 0 | 0 | 0 | 0 | 0 | 0 | 0 | 0 | 0 | 0 | 0 | 0 |
| *Distomus variolosus* | 0 | 0 | 0 | 0 | 0 | 2 | 0 | 0 | 1 | 0 | 0 | 0 | 0 | 0 | 0 | 0 | 0 | 0 | 0 | 0 | 0 | 0 | 0 | 0 | 0 | 0 | 0 | 0 | 0 | 0 | 0 | 0 |
| *Styela plicata* | 3 | 1 | 3 | 0 | 1 | 2 | 2 | 0 | 0 | 0 | 3 | 2 | 1 | 0 | 3 | 3 | 2 | 1 | 3 | 3 | 0 | 0 | 1 | 0 | 3 | 3 | 0 | 0 | 3 | 3 | 2 | 0 |
| *Styela canopus* | 0 | 0 | 0 | 0 | 0 | 0 | 1 | 0 | 0 | 0 | 0 | 0 | 0 | 0 | 0 | 0 | 0 | 0 | 0 | 0 | 0 | 0 | 0 | 0 | 0 | 0 | 1 | 0 | 0 | 0 | 0 | 0 |
| *Polycarpa pomaria* | 0 | 0 | 0 | 0 | 0 | 0 | 0 | 0 | 1 | 0 | 0 | 0 | 0 | 0 | 0 | 0 | 0 | 0 | 0 | 0 | 0 | 0 | 0 | 0 | 0 | 0 | 1 | 0 | 0 | 0 | 0 | 0 |
| *Polycarp asp*. | 0 | 0 | 0 | 0 | 0 | 0 | 0 | 0 | 0 | 0 | 0 | 0 | 0 | 0 | 0 | 0 | 0 | 0 | 0 | 0 | 0 | 0 | 0 | 0 | 0 | 0 | 1 | 0 | 0 | 0 | 0 | 0 |
| *Molgula bleizi* | 0 | 0 | 0 | 0 | 0 | 1 | 1 | 0 | 0 | 0 | 0 | 0 | 0 | 0 | 0 | 0 | 0 | 0 | 0 | 0 | 0 | 0 | 0 | 0 | 0 | 0 | 0 | 0 | 0 | 0 | 0 | 0 |
| *Molgula occidentalis* | 0 | 0 | 0 | 0 | 0 | 0 | 1 | 0 | 0 | 0 | 0 | 0 | 0 | 0 | 0 | 0 | 0 | 0 | 0 | 0 | 0 | 0 | 0 | 0 | 0 | 0 | 0 | 0 | 0 | 0 | 0 | 0 |
| *Molgula* sp. | 0 | 0 | 0 | 0 | 0 | 0 | 0 | 0 | 0 | 0 | 0 | 0 | 0 | 0 | 0 | 1 | 0 | 0 | 0 | 0 | 0 | 0 | 0 | 0 | 0 | 0 | 0 | 0 | 0 | 0 | 0 | 0 |
| *Microcosmus squamiger* | 0 | 2 | 3 | 0 | 1 | 3 | 1 | 0 | 3 | 2 | 2 | 3 | 0 | 1 | 2 | 3 | 0 | 3 | 3 | 2 | 3 | 0 | 2 | 0 | 0 | 1 | 1 | 3 | 2 | 3 | 1 | 2 |
| *Pyura dura* | 0 | 0 | 0 | 0 | 0 | 0 | 0 | 0 | 0 | 0 | 0 | 1 | 0 | 0 | 1 | 0 | 0 | 0 | 0 | 0 | 0 | 0 | 1 | 0 | 0 | 0 | 1 | 0 | 0 | 0 | 0 | 0 |
| *Pyura squamulosa* | 0 | 0 | 0 | 0 | 0 | 0 | 0 | 0 | 0 | 0 | 0 | 0 | 0 | 0 | 0 | 0 | 0 | 1 | 0 | 1 | 0 | 0 | 0 | 0 | 0 | 0 | 0 | 0 | 0 | 0 | 0 | 0 |
| *Ascidia virginea* | 1 | 0 | 0 | 0 | 0 | 0 | 0 | 0 | 0 | 0 | 0 | 0 | 0 | 0 | 0 | 0 | 0 | 0 | 0 | 0 | 0 | 0 | 0 | 0 | 0 | 0 | 0 | 0 | 0 | 0 | 0 | 0 |
| *Ascidia* sp. | 0 | 0 | 0 | 0 | 0 | 0 | 0 | 0 | 0 | 0 | 0 | 0 | 0 | 0 | 0 | 0 | 0 | 0 | 0 | 0 | 0 | 0 | 0 | 0 | 0 | 1 | 0 | 0 | 0 | 0 | 0 | 0 |
| *Ascidiella aspersa* | 2 | 1 | 3 | 0 | 1 | 2 | 0 | 2 | 2 | 1 | 2 | 3 | 0 | 3 | 3 | 1 | 3 | 2 | 2 | 0 | 3 | 2 | 2 | 2 | 2 | 2 | 2 | 1 | 2 | 1 | 0 | 1 |
| *Ascidiella scabra* | 0 | 0 | 0 | 1 | 0 | 1 | 0 | 0 | 0 | 1 | 0 | 0 | 0 | 0 | 0 | 0 | 0 | 0 | 0 | 0 | 1 | 0 | 0 | 0 | 0 | 0 | 0 | 1 | 0 | 0 | 0 | 0 |
| *Phallusia ingeria* | 0 | 0 | 0 | 0 | 0 | 1 | 0 | 0 | 0 | 0 | 0 | 0 | 0 | 0 | 0 | 0 | 0 | 0 | 0 | 0 | 0 | 0 | 0 | 0 | 0 | 0 | 0 | 0 | 0 | 0 | 0 | 0 |
| *Phallusia* sp. | 0 | 0 | 0 | 0 | 0 | 0 | 0 | 0 | 0 | 0 | 0 | 0 | 0 | 1 | 0 | 0 | 0 | 0 | 0 | 0 | 0 | 0 | 0 | 0 | 0 | 0 | 0 | 0 | 0 | 0 | 0 | 0 |
| *Phallusia mammillata* | 0 | 0 | 0 | 0 | 0 | 0 | 0 | 0 | 0 | 0 | 0 | 0 | 0 | 0 | 0 | 0 | 0 | 0 | 0 | 0 | 0 | 0 | 0 | 0 | 0 | 0 | 0 | 0 | 0 | 1 | 0 | 0 |
| *Phallusia fumigate* | 0 | 0 | 0 | 0 | 0 | 0 | 0 | 0 | 0 | 0 | 0 | 0 | 0 | 0 | 0 | 0 | 0 | 0 | 0 | 0 | 0 | 0 | 0 | 0 | 0 | 0 | 0 | 0 | 0 | 0 | 1 | 0 |
| *Ciona intestinalis* | 0 | 2 | 3 | 0 | 3 | 2 | 3 | 3 | 1 | 1 | 3 | 2 | 3 | 2 | 3 | 3 | 2 | 1 | 2 | 1 | 3 | 2 | 1 | 2 | 0 | 3 | 2 | 0 | 3 | 1 | 1 | 1 |
| *Ciona* sp. | 0 | 0 | 0 | 0 | 0 | 0 | 1 | 0 | 0 | 0 | 0 | 0 | 0 | 0 | 0 | 0 | 0 | 0 | 0 | 0 | 0 | 0 | 0 | 0 | 0 | 0 | 0 | 0 | 0 | 0 | 0 | 0 |

**Appendix 1.** Remarks on the assignment of species as native, introduced or cryptogenic.

We considered as native the species reported in the Mediterranean Sea that live usually on natural substrata and are either found only in the Mediterranean Sea or have an Atlanto-Mediterranean distribution (Coll et al. 2010). This accounts for 15 species in the present study (Table 2). Taxonomic remarks on these species and distributional data can be found in Turon (1987), Coll et al. (2010) and Moreno et al. (2014). Remarks on species identified as introduced or cryptogenic follow.

As introduced species we listed eight species:

1. *Clavelina oblonga* is a tropical W Atlantic species that has colonized Brazil and the E Atlantic (Açores, Cape Vert, Senegal) (Rocha et al 2012). *C. oblonga* has been recently reported in the Mediterranean in the Ebro Delta (the same zone where it has been found in this work) by Ordóñez (2013). This species has likely been introduced associated with bivalve aquaculture.
2. *Clavelina lepadiformis* was described in the NE Atlantic. This species has recently been found in Açores, Madeira, South Africa, NW Atlantic and NW Pacific (Monniot et al. 2001; Pyo et al. 2012, Reinhardt et al. 2010; Wirtz 1998). In the Atlanto-Mediterranean region, studies using genetic markers have shown that one lineage inhabiting marinas and artificial substrata in the Mediterranean Sea is in fact an Atlantic clade (likely a cryptic species) introduced to these environments (Tarjuelo et al. 2001; Turon et al. 2003).
3. *Diplosoma listerianum* is a complex of cryptic species, present in all seas except the Antarctic (Locke 2009). One of these species, the so-called Clade A in Pérez-Portela et al. (2013), is native to the Atlantic shores (Berrill 1950) and has been introduced in many areas of the world, including the Mediterranean.
4. *Aplidium accarense* is reported here for the first time in the Mediterranean Sea. This species was described from W Africa. A detailed report on this species is given in Appendix 2.
5. *Ascidiella aspersa* was described in the NE Atlantic Ocean and is common in Atlantic European shores (Gittenberg 2009) from where it has spread to other areas such as the Mediterranean, Western Atlantic (North and South), South Africa, India, and North and South Pacific (Callahan et al. 2010; Locke 2009; Tatián et al. 2010; Nishikawa et al 2014). In the Mediterranean Sea, it is an alien species confined to harbors and artificial environments (Turon 1988, Nishikawa et al 2014).
6. *Polyandrocarpa zorritensis* is a species described from Perú (Van Name 1931) and found afterwards in Brazil (Millar 1958). It has been introduced since the seventies in the Mediterranean Sea, where it has been reported in enclosed environments in Italy (Brunetti 1978, Brunetti and Mastrototaro 2004). It was reported in the same area here found by Turon and Perera (1988).
7. *Styela plicata* is a cosmopolitan ascidian whose origin, although not clearly defined as yet, is considered to be the NW Pacific (Barros et al. 2009; Carlton 2009). This species was described along the Northeast coast of North America from a specimen attached to a ship’s hull (Van Name 1945) and is highly likely to be an old introduction (Carlton 2009). It is now common in tropical and temperate waters worldwide (Locke 2009; Pineda et al. 2011).
8. *Microcosmus squamiger* is a species of Australian origin that has spread worldwide in temperate areas (Rius et al. 2012). In the Mediterranean Sea, *M. squamiger* has been observed since the sixties (wrongly identified as *M. exasperatus* in earlier works) and is abundant in confined environments in western Mediterranean, where it also occurs on natural substrata (Turon et al. 2007).

Following Carlton (2000, 2009), we have assigned cryptogenic status to species for which not enough evidence (distributional, genetic, or otherwise) is available to assign a status as native or introduced. In many cases, these are cosmopolitan species with disjoint distributions inhabiting mostly or exclusively artificial habitats in their distribution range; these species are likely to be old introductions (e.g. centuries) that have been shuffled repeatedly between oceans and whose native range cannot be discerned. As cryptogenic species we listed five species:

1. *Ciona intestinalis* is considered a species complex that includes several cryptic species (Caputi et al. 2007; Iannelli et al. 2007; Nydam and Harrison 2007). Recently, Zhan et al., (2010) found evidence for the existence of four major phylogroups (named sp. A to sp. D). While sp. C was restricted to the Mediterranean Sea and sp. D to the Black Sea, the two invasive species (sp. A and sp. B) have disjoint distributions and no genetic structure was found within them in spite of the use of several markers. The native ranges of this species complex remain highly debated (Therriault and Herborg 2008; Zhan et al. 2010).
2. *Botryllus schlosseri* has been described from specimens found in an English harbour (Berrill 1950) and is now widespread in warm and temperate waters across both hemispheres (Locke 2009). Its native range is under debate. Berrill (1950) is often cited as suggesting a Mediterranean origin (e.g. Stoner et al. 2002) although Berrill’s monograph only indicates that Europe is the likely source of introductions along the American coastline. López-Legentil et al., (2006) found that the genetic structure of populations from the western Mediterranean Sea was not consistent with a native species and suggested a Pacific origin, following what Carlton (2005) had previously suggested. On the other hand, Ben-Shlomo et al., (2006) found evidence that the species was already present along the European Atlantic coasts during the last glaciations, and Lejeusne et al., (2011) rejected the Pacific origin of the species hypothesis. Thus, based on current data it seems advisable to consider the species as cryptogenic for the time being.
3. *Botrylloides leachi* was described in the Mediterranean Sea, and can be found all along European shores, as well as in South Africa, Australia and the Western Pacific (Locke 2009). It might be of Mediterranean origin (Berrill 1950) but, according to Carlton (2005), it could have been introduced to the Mediterranean Sea as early as the 1500s from the Pacific Ocean, the centre of Botryllid diversity.
4. *Ascidiella scabra* is a species described in NE Atlantic, which has been occasionally regarded as synonymous with *A. aspersa*. Recent molecular work confirms that it is a valid species with an invasive potential (Nishikawa et al 2014). Nevertheless, the Mediterranean specimens here analysed formed a genetic cluster distinct from that of Atlantic *A. scabra* and from Atlantic and Mediterranean *A. aspersa* (Fig. S3). Until more genetic evidence becomes available, we are unable to assign a status to the Mediterranean specimens of *A. scabra*.
5. *Styela canopus* has a worldwide distribution in tropical and temperate regions (Locke 2009). In the Mediterranean it has been traditionally referred to as *Styela partita* (Turon 1987). Detailed genetic and phylogeographic studies for this species are needed, and until then *S. canopus* status in the Mediterranean Sea is better left as cryptogenic.

Literature cited

Barros RC, Rocha RM, Pie MR (2009) Human-mediated global dispersion of *Styela plicata* (Tunicata, Ascidiacea). Aquat Invasions 4: 45-57

Ben-Shlomo R, Paz G, Rinkevich B (2006) Postglacial-period and recent invasions shape the population genetics of Botryllid ascidians along European Atlantic coasts. Ecosystems 9: 1118-1127

Berrill NJ (1950) The Tunicata with an account of the British species. Ray Society, London

Brunetti R (1978) *Polyandrocarpa zorritensis* (Van Name, 1931). A colonial ascidian new to the Mediterranean record. Vie Milieu 28-29: 647-652

Brunetti R, Mastrototaro F (2004) The non-indigenous stolidobranch ascidian Polyandrocarpa zorritensis in the Mediterranean: description, larval morphology and pattern of vascular budding. Zootaxa 528: 1-8

Callahan AG, Deibel D, McKenzie CH, Hall JR, Rise ML (2010) Survey of harbours in Newfoundland for indigenous and non-indigenous ascidians and an analysis of their cytochrome c oxidase I gene sequences. Aquat Invasions 5: 31-39

Caputi L, Andreakis N, Mastrototaro F, Cirino P, Vassillo M, Sordino P (2007) Cryptic speciation in a model invertebrate chordate. Proc Nat Acad Sci USA 104: 9364-9369

Carlton JT (2000) Quo vadimus exotica oceanica? Marine bioinvasion ecology in the twenty-first century. Marine Bioinvasions: Proceedings of the First National Conference (ed. by J. Pederson), pp. 6-23. Massachusetts Institute of Technology Sea Grant College Program, Cambridge

Carlton JT (2005) Setting ascidian invasions on the global stage. *Proceedings of the First International Invasive Sea Squirt Conference*. Woods Hole Oceanographic Institution, Woods Hole, Massachusetts

Carlton JT (2009) Deep Invasion Ecology and the Assembly of Communities in Historical Time. Biological Invasions in Marine Ecosystems (ed. by G. Rilov, and J.A. Crooks), pp. 13-56. Springer-Verlag, Berlin, Heidelberg

Coll M, Piroddi C, Steenbeek J, Kaschner K, Ben Rais Lasram F, et al. (2010) The biodiversity of the Mediterranean Sea: Estimates, patterns, and threats. PLoS ONE 5(8): e11842

Gittenberg A (2009) Invasive tunicates on Zeeland and Prince Edward Island mussels, and management practices in The Netherlands. Aquat. Invasions 4: 279-281

Iannelli F, Pesole G, Sordino P, Gissi C (2007) Mitogenomics reveals two cryptic species in *Ciona intestinalis*. Trends Genet 23: 419-422

Lejeusne C, Bock DG, Therriault TW, Macisaac HJ, Cristescu ME (2011) Comparative phylogeography of two colonial ascidians reveals contrasting invasion histories in North America. Biol Invasions 13: 635-650

Locke A (2009) A screening procedure for potential tunicate invaders of Atlantic Canada. Aquat Invasions 4: 71-79

López-Legentil S, Turon X, Planes S (2006) Genetic structure of the star sea squirt, *Botryllus schlosseri*, introduced in southern European harbours. Mol Ecol 15: 3957-3967

Monniot C (1962) Les *Microcosmus* des cotes de France. Vie Milieu 12: 397-432

Monniot C, Monniot F, Griffiths CL, Schleyer M (2001) South African ascidians. Ann S Afric Mus 108: 1-141

Moreno TR, De Faria SB, Rocha RM (2014) Biogeography of Atlantic and Mediterranean ascidians. Mar Biol 161: 2023-2033

Nishikawa T, Oohara I, Saitoh K, Shigenobu Y, Hasegawa N, Kanamori M, Baba K, Turon X, Bishop JDD (2014) Molecular and morphological discrimination between and invasive ascidian, *Ascidiella aspersa* and its congener *A. scabra* (Urochordata: Ascidiacea). Zool Sci 31: 180-185

Nydam ML, Harrison RG (2007) Genealogical relationships within and among shallow-water *Ciona* species (Ascidiacea). Mar Biol 151: 1839-1847

Ordóñez V (2013) Ecology and genetics of invasive ascidians in western Mediterranean. PhD dissertation, University of Barcelona, Barcelona

Pérez-Portela R, Arranz V, Rius M, Turon X (2013) Cryptic speciation or global spread? The case of a cosmopolitan ascidian with limited dispersal capabilities. Sci Rep 3: 3197

Pineda MC, López-Legentil S, Turon X (2011) The whereabouts of an ancient wanderer: Global phylogeography of the solitary ascidian *Styela plicata*. PLoS One 6: e25495

Pyo J, Lee T, Shin S (2012) Two newly recorded alien ascidians (Chordata, Tunicata, Ascidiacea) based on morphological and molecular phylogenetic analysis in Korea. Zootaxa 3368: 211-228

Reinhardt JF, Stefaniak LM, Hudson DM, Mangiafico J, Gladych R, Whitlatch RB (2010) First record of the non-native light bulb tunicate *Clavelina lepadiformis* (Müller, 1776) in the northwest Atlantic. Aquat Invasions 5: 185-190

Rius M, Turon X, Ordóñez V, Pascual M (2012) Tracking invasion histories in the sea: facing complex scenarios using multilocus data. PLoS One 7: e35815

Stoner DS, Ben-Shlomo R, Rinkevich B, Weissman IL (2002) Genetic variability of *Botryllus schlosseri* invasions to the east and west coasts of the USA. Mar Ecol Prog Ser 243: 93-100

Tarjuelo I, Posada D, Crandall K, Pascual M, Turon X (2001) Cryptic species of *Clavelina* (Ascidiacea) in two different habitats: harbours and rocky littoral zones in the northwestern Mediterranean. Mar Biol 139: 1432-1793

Tatián M, Schwindt E, Lagger C, Varela MM (2010) Colonization of Patagonian harbours (SW Atlantic) by an invasive sea squirt (Chordata, Ascidiacea). Spixiana 33: 111-117

Therriault TW, Herborg LM (2008) A qualitative biological risk assessment for vase tunicate *Ciona intestinalis* in Canadian waters: using expert knowledge. ICES J Mar Sci 65: 781-787

Turon X (1987) Ascidians from the shores of Catalonia and Balearic Islands. PhD dissertation, University of Barcelona, Barcelona

Turon X (1988) Distribución ecológica de las ascidias en las costas de Cataluña e Islas Baleares (Mediterráneo occidental). Misc Zool 12: 219-236

Turon X, Perera M (1988) Las ascidias del Delta del Ebro. Aspectos faunísticos y cuantitativos. Publ Dept Zool Univ Barcelona 14:81-90

Turon X, Nishikawa T, Rius M (2007) Spread of *Microcosmus squamiger* (Ascidiacea: Pyuridae) in the Mediterranean Sea and adjacent waters. J Exper Mar Biol Ecol 342: 185-188

Turon X, Tarjuelo I, Duran S, Pascual M (2003) Characterising invasion processes with genetic data: an Atlantic clade of *Clavelina lepadiformis* (Ascidiacea) introduced into Mediterranean harbours. Hydrobiologia 503: 29-35

Van Name WG (1931) New North and South American ascidias. Bull Am Mus Nat Hist 61: 207-225

Van Name WG (1945) The North and South American ascidians. Bull Am Mus Nat Hist 84: 1-476

Wirtz P (1998) Twelve invertebrate and eight fish species new to the marine fauna of Madeira, and a discussion of the zoogeography of the area. Helg Meeres 52: 197-207

Zhan A, Macisaac HJ, Cristescu ME (2010) Invasion genetics of the *Ciona intestinalis* species complex: from regional endemism to global homogeneity. Mol Ecol 19: 4678-4694

**Appendix 2.** Taxonomic remarks on *Aplidium accarense.*

Colonies encrusting to cushion-shaped, to 7 cm in diameter and 1.5 cm in thickness. Color usually yellowish due to yellow zooids on a darker background of translucent tunic (Fig S1-X). Zooid systems circular or elongated, and discernible only on living material. The tunic is soft, translucent, without encrusted sand (albeit some fecal pellets occasionally embedded in it).

The zooids (Fig. 1A) vary in length from 2 to 10 mm depending on the degree of contraction. They are yellowish in color but the incubated embryos can be yellow to bright orange. Oral siphon with 6 lobes, atrial aperture at the level of the first row of stigmata surmounted by a short and simple atrial languet. Number of stigmata rows between 6 and 9, being 7 the most common (Fig. 1B). The number of stigmata per half row is ca. 12 in the middle of the branchial sac. The digestive system comprises a stomach with up to 17 folds (7-8 in the visible sector), followed by a short post-stomach and a dilated mid-intestine lying at the bottom of the intestinal loop. The rectum has two broad and small proximal caeca and ends in an anus situated mid-way up the branchial sac (at the level of the 4^th^ stigmata row).

The post-abdomen has well-developed gonads in the colonies examined. The ovary is formed by a few oocytes. In well-relaxed zooids they are at some distance from the intestinal loop. Most of the post-abdomen is occupied by densely packed male follicles (Fig. 1A) and a filled sperm duct passes anteriorly towards the peribranchial space. There are embryos incubating in the right part of the peribranchial cavity. They number from 4 to 15 but only the 4-5 anterior most embryos are well-developed larvae. When there are many embryos the less mature protrude from the peribranchial cavity forming a posterolateral pouch (Fig. 1A). Fully developed larvae are 0.4 to 0.6 mm in trunk length (Fig. 1C). They have three sagittal adhesive papillae and spherical ectodermal ampullae, apparently unstalked, forming four groups: two between the papillae and one each dorsally and ventrally. These ampullae are variable in number (>25) and arrangement, but in less mature larvae (Fig. 1D) they are less numerous and form two rows of ca. 8-10 ampullae each.

Additional remarks

The characteristics of the material examined agree well with previous descriptions of the species (Millar 1953, Monniot 1969, Rocha et al 2005), although with some variability as for the colonies’ color. The shape of the atrial siphon, branchial sac (the number of stigmata rows reported for the species ranges between 6 and 10), and the stomach, digestive loop and anus position all agree with previous descriptions. The species is reported to incubate larvae in the right peribranchial cavity, and the larvae are characteristic in having ca. 10 ectodermal ampullae at each side (Millar 1953, Monniot 1969). This is the character observed in near-mature larvae (Fig. 1D), although the number of ampullae increases in fully mature larvae (Fig. 1C).

This is the first record of this species in the Mediterranean. *Aplidium accarense* was described by Millar (1953) from W Africa (Gold Coast), and was later reported from Cape Verde Islands (Monniot & Monniot 1967) and Senegal (Monniot 1969, Lafargue & Wahl 1987). The species has been found recently in Southern Brazil (Rocha et al 2005, Rocha & Bonnet 2009) and, although classified as cryptogenic in that area, it seems highly likely that it has been introduced in W Atlantic (Rocha & Bonnet 2009). In the Mediterranean, aside from the present record, the species is abundant in harbors and lagoons of S Italy (Naples region, XT pers. obs.), so it is likely that *A. accarense* is undergoing an expansion in the Mediterranean, where they may constitute a pest in the near future.

Literature cited

Lafargue F, Wahl M (1987) Contribution to the knowledge of littoral ascidians (Ascidiacea, Tunicata) of the Senegalese coast. Bull Inst Fond Afrique Noire 46, Sér A (3-4): 385-402

Millar RH (1953) On a collection of ascidians from the Gold Coast. Proc Zool Soc Lond 123: 277-325

Monniot C, Monniot F (1967) Tuniciers benthiques. Campagnes de la Calypso aux Iles du Cap Vert (1959). Ann Inst Océanogr, Monaco 45(2): 3-18

Monniot F (1969) Sur une collection d’ascidies composes de Dakar. Bull Mus natn Hist Nat, Paris, 2e Sér, 41(2): 426-45

Rocha RM, Bonnet NYK (2009) Ascídias (Tunicata, Ascidiacea) introduzidas no Arquipélago de Alcatrazes, São Paulo. Iheringia, Sér Zool, Porto Alegre 99(1): 27-35

Rocha RM, Montero TR, Metri R (2005) Ascídias (Tunicata, Ascidiacea) da Reserva Biológica Marinha do Arvoredo, Santa Catarina, Brasil. Rev Brasil Zool 22(2): 461-476


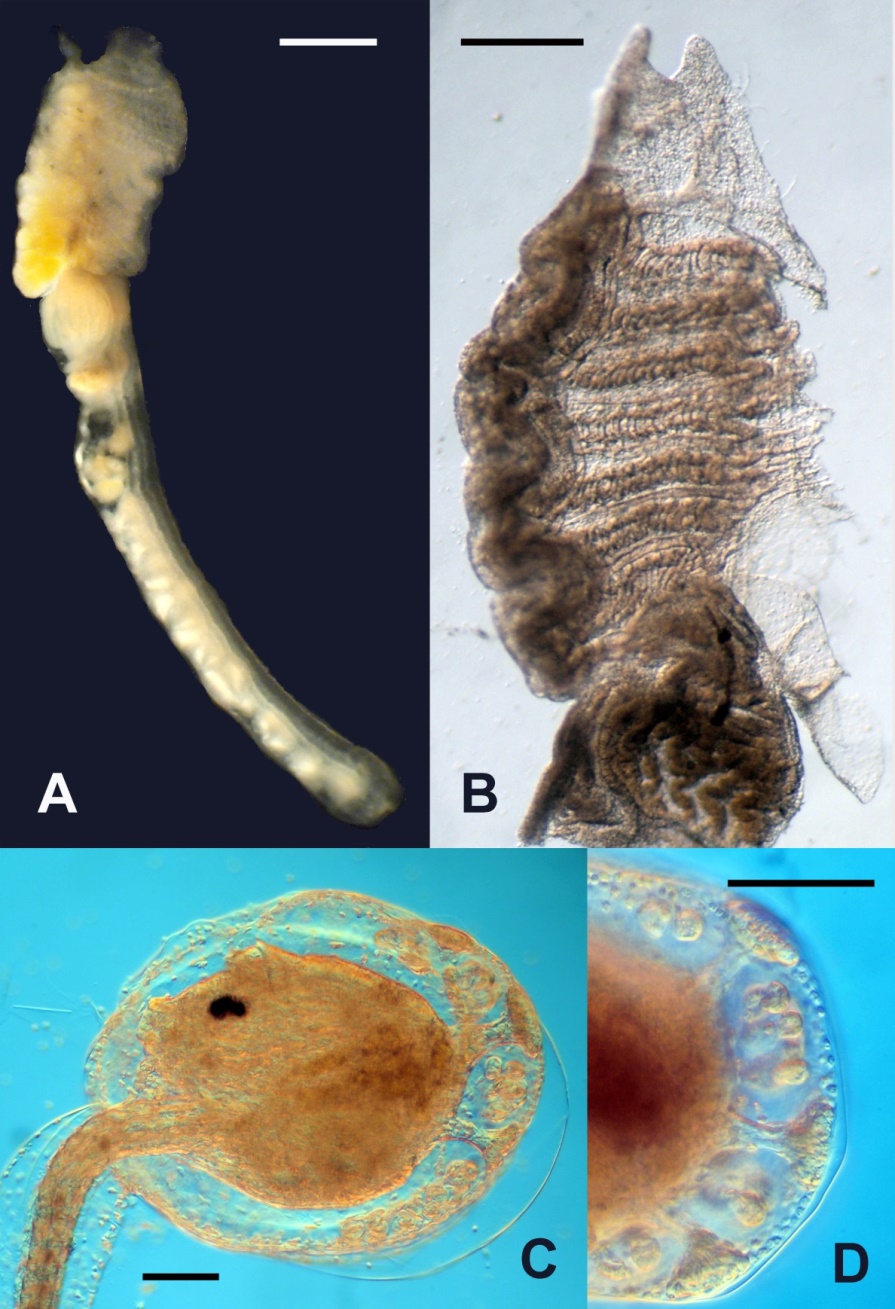


Figure 1. (A) Whole zooid with gonads and incubating larvae. (B) Sagitally cut anterior region of a zooid. (C) Lateral view of a fully mature larva. (D) anterior part of a less developed larva showing adhesive papillae and ectodermal ampullae. Scale bars: (A) 1 mm; (B) 0.5 mm; (C) 0.1 mm; (D) 0.1 mm**Supplementary Figures**

**Figure S1.** Selected images of ascidians in Catalan harbors. (A) *Ascidiella aspersa* (Müller, 1776), (B) *Ascidiella scabra* (Müller, 1776), (C) *Phallusia ingeria* Traustedt, 1883, (D) *Phallusia mammillata* (Cuvier 1815), (E) *Phallusia fumigata* (Grube, 1864), (F) *Ciona intestinalis* (Linnaeus, 1767), (G) *Botryllus schlosseri* (Pallas, 1766), (H) *Botrylloides leachii* (Savigny, 1816), (I) *Polyandrocarpa zorritensis* (Van Name, 1931), (J) *Distomus variolosus* Gaertner 1774, (K) *Styela plicata* (Lesueur, 1823), (L) *Styela canopus* (Savigny, 1816), (M) *Polycarpa pomaria* (Savigny, 1816), (N) *Molgula bleizi* (Lacaze-Duthiers, 1877), (O) *Molgula occidentalis* Traustedt, 1883, (P) *Microcosmus squamiger* Michaelsen, 1927, (Q) *Pyura dura* (Heller, 1877), (R) *Pyura squamulosa* (Alder, 1823), (S) *Diplosoma listerianum* (Milne-Edwards, 1841), (T) *Diplosoma spongiforme* (Giard, 1872), (U) *Trididemnum cereum* (Giard, 1872), (V) *Didemnum fulgens* (Milne-Edwards, 1841), (W) two color morphs of *Morchellium argus* (Milne-Edwards, 1841), (X) *Aplidium accarense* (Millar, 1953), (Y) *Clavelina oblonga* (Herdman, 1880), and (Z) *Clavelina lepadiformis* (Müller, 1776).

**Figure S2**. Linear regression between harbor size (linear length of all docks) and species richness.

**Figure S3.** Neighbor-joining tree based on the Kimura 2-parameter distances combining *Ascidiella* sequences of Nishikawa et al. (2014) (haplotype codes named as in that work) with our dataset for this genus (represented by GenBank accession numbers). A sequence of *Ascidia zara* from Nishikawa et al. (2014) was used as outgroup. Note that, as our sequences had to be trimmed to fit those of the previous work, some of them resulted in the same haplotype. Nodes with >80% bootstrap support are indicated. Analyses were performed in Mega v6 software (Tamura et al. 2013).

**Figure S4.** Non-metric MDS configurations as in Fig. 4 with indication of the codes of the harbors (as in Table 1).

**Figure S1**

**
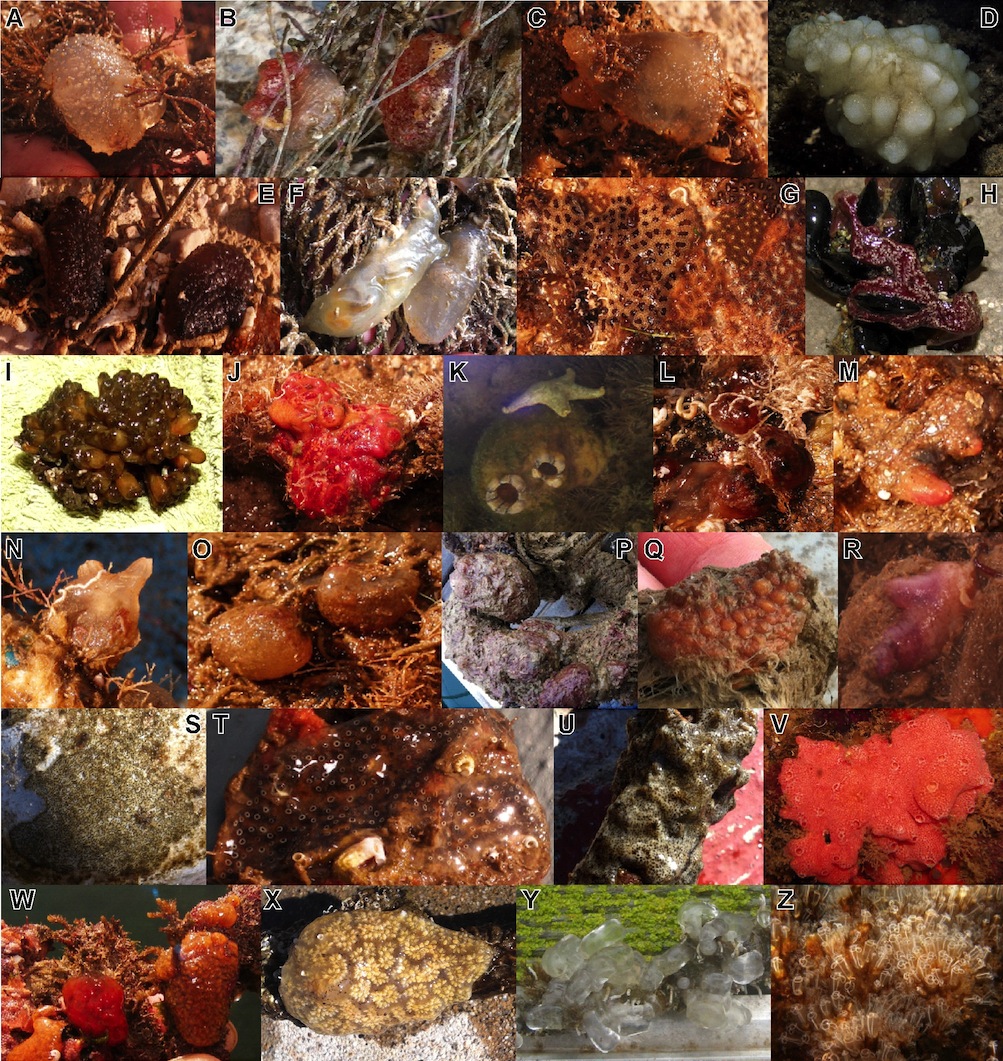
**

**Figure S2**

**Figure S3**

**Figure S4**
